# Effect of saponins from *gynostemma pentaphyllum* on iron metabolism in apolipoprotein E deficient mice

**DOI:** 10.1186/s40001-026-03871-6

**Published:** 2026-01-12

**Authors:** Tingyun Huang, Xin Xiao, Jimin Ma, Junwen Fang, Yuxin Bao

**Affiliations:** 1 First Clinical College, Zunyi Medical University, Zunyi, 563000 Guizhou China; 2 The Second Affiliated Hospital of Zunyi Medical University, Zunyi, 563000 Guizhou China; 3 Institute of Life Sciences, Zunyi Medical University, No. 6, Xuefu West Road, Xinpu New District, Zunyi, 563000 Guizhou China; 4 College of Basic Medical Sciences, Zunyi Medical University, Zunyi, 563000 Guizhou China

**Keywords:** Apolipoprotein E knock-out (*ApoE* KO/*ApoE*^*−/−*^) mice, *Gynostemma Pentaphyllum* (GP), Gypenosides (GPS), Iron metabolism

## Abstract

**Background:**

*Gynostemma pentaphyllum* (GP) is known as the “elixir of life” in Guizhou Province, China, as it has been widely consumed by the elderly. Numerous studies have shown that gypenosides (GPS) extracted from GP are involved in lipid metabolism. Apolipoprotein E (*ApoE*) is a polymorphic protein with multiple biological functions, such as regulating lipid transport and iron metabolism. The deficiency of *ApoE* can lead to disorders in both lipid and iron metabolism. Therefore, *ApoE* knockout (*ApoE*^*−/−*^) mice are widely used in the research of disease models related to lipid and iron metabolism. It has been found through research that GPS ameliorates *ApoE* deficiency-induced dyslipidemia, while our prior research has established *ApoE* as indispensable for maintaining systemic iron homeostasis. According to the pharmacological effects of GPS, they can regulate lipid metabolism through pathways such as anti-inflammation and oxidative stress. These pathways also play a crucial role in the body’s iron metabolism. Thus, this paper hypothesizes that GPS can reverse the abnormal iron metabolism caused by *ApoE* deficiency. It further explores the impact of GPS on iron metabolism and the underlying mechanism, aiming to provide a theoretical basis for the development of drugs that regulate iron homeostasis.

**Methods:**

We randomly divided C57BL/6 mice were randomly divided into blank group (WT), apolipoprotein E knockout group (*ApoE* KO/*ApoE*^*−/−*^) and gypenosides group (*ApoE*^*−/−*^ + GPS). The serum iron content, tissue iron content, transferrin receptor 1 (TfR1), ferroportin 1 (Fpn1), divalent metal transporter 1 (DMT1), iron regulatory proteins (IRPs), ferritin light chain (FTL), ferritin heavy chain (FTH), nuclear factor erythroid 2-related factor 2 (NRF2) and hepcidin expression in liver and spleen of the three groups of mice were studied.

**Results:**

The results demonstrate that gypenosides reduce *ApoE* deficiency-induced iron accumulation by downregulating TfR1 (a cellular iron import protein) and upregulating Fpn1 (an iron export protein). In the spleen of *ApoE*^*−/−*^ mice, this regulation occurs through Nrf2-dependent upregulation of Fpn1 and IRP2-mediated downregulation of TfR1, whereas in the liver, neither Nrf2 nor IRPs play a dominant role in the altered expression of TfR1 and Fpn1 induced by *ApoE* knockout.

**Conclusions:**

Gypenosides can reduce tissue iron accumulation in the liver and spleen of *ApoE*-deficient mice, suggesting that, based on its function in regulating lipid metabolism, gypenosides also possess the potential ability to regulate iron metabolism.

**Supplementary Information:**

The online version contains supplementary material available at 10.1186/s40001-026-03871-6.

## Introduction

*Gynostemma pentaphyllum* (GP) is commonly known as the “elixir of life” in Guizhou Province, China, as it has been widely consumed by the elderly. Moreover, it is a plant with both medicinal and edible properties, traditionally used in folk medicine to treat various diseases, including diabetes, metabolic syndrome, aging, neurodegenerative diseases, and cancer [1]. The phytochemical profile of *G. pentaphyllum* encompasses gypenosides (GPS), polysaccharides (GPP), flavonoids, and phytosterols, with GPS being the most extensively studied bioactive constituents [2]. Numerous studies have confirmed that GPS can regulate systemic lipid metabolism [3–5]. The organism, as a precisely regulated entity, maintains systemic homeostasis under physiological conditions. Studies have revealed that lipid metabolism and iron metabolism share common regulatory hubs, such as inflammatory stimuli (IL) [6], oxidative stress (HIF) [7, 8], nutrient integration (mTOR) [9], and energy metabolism (AMPK) [9] pathways.

Iron (Fe), an essential trace metal, plays a pivotal role in the catalytic centers of diverse enzymes and oxygen-carrier proteins [10]. The transport, storage, and regulation of iron are tightly controlled at both cellular and systemic levels to maintain its concentration within an optimal physiological range [11]. Extensive evidence confirms that disruptions in iron homeostasis—including iron deficiency and overload—cause multifaceted harm to the organism. Perturbations in iron homeostasis significantly elevate the risk of cancer, diabetes, neurodegenerative diseases, and cardiovascular disorders [12–15]. Recent studies have clearly shown that diseases caused by disorders of iron metabolism are far more widespread than previously imagined. There are hundreds of millions of diseases associated with iron metabolism disorders, which may be the diseases that affect the largest number of people [16, 17].

Apolipoprotein E (*ApoE*), a 34 kDa glycoprotein composed of 299 amino acids, plays a critical role in hepatic and extrahepatic uptake of plasma lipoproteins and cholesterol efflux from lipid-laden macrophages [18]. In humans, the *ApoE* protein exists in three structurally distinct isoforms—*ApoE2, ApoE3* and *ApoE4*—encoded by three alleles (*ε2*, *ε3*, and *ε4*). Murine *ApoE* shares functional and regional similarities with human *ApoE3* [19]. *ApoE* is recognized as a key apolipoprotein in lipoprotein metabolism, regulating plasma clearance of lipoproteins and maintaining homeostasis of plasma lipid levels and tissue lipid content [20, 21]. *ApoE* deficiency leads to metabolic disorders associated with lipid dysregulation [22–24]. Consequently, *ApoE* knockout (*ApoE*^*−/−*^) mice are widely employed as models for studying lipid metabolism abnormalities. Emerging evidence further indicates that *ApoE* is implicated in systemic iron regulation, with elevated iron levels linked to atherosclerosis and neurodegenerative diseases [25]. Our prior studies demonstrated that *ApoE* deficiency induces age-dependent progressive iron accumulation in murine tissues [26], a finding corroborated by recent reports of cerebral iron overload and neuronal damage in *ApoE*^*−/−*^ mice [27]. These collective findings underscore *ApoE*’s dual role in modulating both lipid metabolism and iron homeostasis. Building on this evidence, we hypothesized that gypenosides (GPS)—a traditional herbal compound clinically utilized for its hypolipidemic properties—may also influence iron metabolism in *ApoE*^*−/−*^ mice. To validate this hypothesis, we measured major parameters involved in iron metabolism (ferritins, TfR1, Fpn1, DMT1, IRPs, hepcidin, Nrf2), as they underlie the maintenance of iron homeostasis. First, we quantified serum and tissue iron levels, as well as the expression of ferritin light chain (FTL) and heavy chain (FTH) in the liver and spleen of homozygous *ApoE*^*−/−*^ knockout mice, wild-type (WT, *ApoE⁺/⁺*) mice, and *ApoE*^*−/−*^ mice treated with GPS (*ApoE*^*−/−*^ + GPS). We then analyzed expression changes in key iron regulatory proteins: transferrin receptor 1 (TfR1), the primary cellular iron importer, and ferroportin 1 (Fpn1), the sole known iron exporter. Finally, we investigated critical molecular regulators of TfR1 and Fpn1, including iron regulatory proteins (IRPs), hepcidin, and nuclear factor erythroid 2-related factor 2 (Nrf2). Our study provides the first evidence that GPS exert iron-regulatory effects in *ApoE*^*−/−*^ mice, effectively reducing hepatic and splenic iron content.

## Materials and methods

### Materials

Rabbit polyclonal anti-DMT1, anti-FTL, and anti-Nrf2 (Proteintech, USA); anti-Fpn1 (Novus Biologicals, USA); anti-FTH (Bioworld Technology, USA); anti-Hepcidin, anti-IRP1, anti-IRP2, and anti-TfR1 (Abcam, UK). HRP-Conjugated Affinipure Goat Anti-Rabbit IgG(H + L) (Proteintech, USA). TRIzol and RIPA lysis buffer (Beyotime Biotechnology, China); cDNA Synthesis Kit and SYBR qPCR Master Mix (Vazyme Biotech, China); GPS (98% purity, Shanxi Zhongxin Biotechnology Co., Ltd., China). Serum Iron Assay Kit (Colorimetric) (Catalog No. A039-1-1) from Nanjing Jiancheng Bioengineering Institute. Total Iron Ion Content Assay Kit for Tissues (Colorimetric) (Catalog No. E1050) from Beijing Pulilai Gene Technology Co., Ltd. Perl’s Iron Stain (Catalog No. G1422) and DAB-enhanced Perls staining (Catalog No. G1428) kits from Beijing Solarbio Science & Technology Co., Ltd. All reagents/supplies were nuclease-free and autoclaved. All solutions were freshly prepared before use.

### Animals, grouping, and GPS treatment

8-week-old specific pathogen-free (SPF) grade ApoE^*−*^/^*−*^ mice and wild-type mice (C57BL/6, male), weighing 20–25 g, were purchased from Jiangsu Jicui Yaokang Biotechnology Co., Ltd. (Jiangsu, China) and Hangzhou Resources Experimental Animal Technology Co., Ltd. (Zhejiang, China), respectively, and their genotypes were verified by PCR (Supplementary Figure S1). After 7 days of acclimatization to the experimental environment, 18 male mice were selected and randomly divided into 3 groups: the WT group, the ApoE^*−*^/^*−*^ group, and the ApoE^*−*^/^*−*^ + GPS group, with 6 mice in each group. Mice in the ApoE^*−*^/^*−*^ + GPS group were orally administered (gavaged with) GPS at a dose of 250 mg/kg, which was suspended in 0.1% carboxymethyl cellulose sodium (CMC-Na) and delivered at a volume of 5 mL/kg. Both the Wild-Type (WT) and ApoE^*−*^/^*−*^ control groups received an equal volume of the vehicle (0.1% CMC-Na) by oral gavage. Gavage was performed once daily at the same time each day for 8 consecutive weeks. Body weight of the mice was monitored every 3 days to avoid significant differences in body weight among individuals. The housing conditions for the mice were strictly controlled as follows: the mice were housed in polycarbonate cages with a maximum density of 5 mice per cage, ensuring each mouse had a minimum floor area of ≥ 150 cm^2^. The animal room was maintained at a temperature of (22 ± 2)℃ and a relative humidity of 60–65%, with a 12-h light/12-h dark cycle (lights on from 07:00 to 19:00) to mimic the natural circadian rhythm. Sterilized corn cob bedding was used during the experiment and changed twice a week to maintain hygiene. Standard rodent chow and autoclaved drinking water were provided ad libitum, with the chow containing approximately 45 mg/kg iron. No adverse events were observed during dynamic monitoring. In this study, all animals, experimental units, and data points from all experimental groups were fully included in the final analysis, with no exclusions. All animals care and experimental protocols were conducted in accordance with the Animal Management Rules of the Ministry of Health of China and were approved by the Animal Ethics Committees of Zunyi Medical University (ZMU21-2503-415).

### Randomisation and blinding

All candidate animals were numbered (1–18), and labels corresponding to these numbers were made. The labels were thoroughly mixed (by placing them in a container and shaking) to ensure they were completely randomized. The required number of labels were selected blindly, and the animals corresponding to the numbers on the selected labels were the chosen individuals. All intervention operations (e.g., gavage, sample collection) for all experimental groups were performed by the same researcher and strictly followed a randomly generated sequence to avoid systematic errors caused by differences in operator proficiency or treatment order. Experimental procedures (e.g., animal weighing) were conducted at the same time period daily to reduce the impact of circadian rhythms on the physiological status of animals. Researchers responsible for sample detection were unaware of the group information corresponding to the samples (samples were only labeled with random numbers) to avoid interference from subjective judgment in result interpretation. Random allocation of cage positions. The number of animals per cage was controlled within 5, and differences in age and body weight were minimized (± 5 g). Animals in each cage were raised under the same environmental conditions (feeding, humidity, temperature, etc.) to reduce the impact of inter-cage environmental differences on inter-group comparisons. The collection methods (e.g., anesthetic dosage, perfusion rate) and storage conditions (e.g., − 80 ℃ freezing time, number of freeze–thaw cycles) for blood and tissue samples were strictly standardized to avoid detection errors caused by differences in sample processing.

### Animal anesthesia and sampling

All mice were sacrificed at 16 weeks of age. Mice were sacrificed using 1% sodium pentobarbital (40 mg/kg body weight, intraperitoneal injection), and according to the ethical guidelines for animal experiments. Fasting blood samples were collected, and the liver and spleen were weighed. All samples were stored in a − 80 °C refrigerator for subsequent detection.

### Serum iron and iron contents in the liver and spleen

The serum iron content was detected by the kit. Total iron concentrations in the spleen and liver were determined using a graphite furnace atomic absorption spectrophotometer (GFAAS).

### Perl’s iron stain and DAB-enhanced perls staining

Paraformaldehyde-fixed paraffin-embedded tissues were sectioned into 3 μm sections and stored at room temperature. Sections were deparaffinized using xylene and then were rehydrated in ethanol. Before use, prepare Perls Stain Working Solution (The volume ratio of Prussian Blue Staining Solution to hydrochloric acid is 1:1), drip cover the sections for staining for 30 min, and soak in distilled water for 10 min. The images from at least four fields each from three mice were examined, and dye with Nuclear Fast Red Solution for 10 min. Rinse fully in distilled water for 5 min. Subsequently, the sample was dehydrated and transparent through a series of ethanol and xylene treatments. Finally, sealed and observed by microscope (Leica, Germany). For DAB-enhanced Perls staining (This method is applicable to iron deficiency samples or non-iron overload model staining), slides were immersed for Perls Working Solution in the ratio of 1: 1 and then stained with DAB. All slides were counterstained with hematoxylin and then visualized using a microscope (Leica, Germany).

### Immunohistochemistry staining

Formalin-fixed paraffin-embedded tissues were sectioned into 3 µm sections and stored at room temperature. Sections were deparaffinized using xylene and then were rehydrated in ethanol before being subjected to heat-activated antigen retrieval in citrate buffer (pH 6). Slides then were blocked for nonspecific binding using protein block, and endogenous peroxidase activity was quenched. Sections were immunohistochemically stained with rabbit anti-TfR1 (1:200), rabbit anti-Fpn1 (1:100) and counterstained with hematoxylin. Signal was detected using a DAB substrate following the manufacturer’s recommendations. The immunostaining images were scanned randomly under a light microscope (Leica, Germany) by a single investigator who was blind to sample identity.

### Immunofluorescence staining

Formalin-fixed paraffin-embedded tissues were sectioned into 3 µm sections and stored at room temperature. Sections were deparaffinized using xylene and then were rehydrated in ethanol before being subjected to heat-activated antigen retrieval in citrate buffer (pH 6). slices were incubated in blocking solution followed by overnight incubation at 4 °C with primary antibodies: rabbit anti-FTL (1:100), rabbit anti-FTH (1:100), and rabbit anti-hepcidin (1:100). After washing with PBST, the slides were incubated with Alexa Fluor 488, conjugated secondary antibodies for 1-h at 37 °C. The nuclei were counterstained with DAPI. Antifluorescence quenching mounting solution was added dropwise to the glass slide and the results were visualized with a confocal microscope (Carl Zeiss; Heidenheim, Germany).

### Real-time PCR analysis

Total RNA was extracted from mouse liver and spleen tissues and reverse-transcribed into cDNA. Gene expression was analyzed by SYBR green PCR using a LightCycler 96 PCR instrument (Roche Diagnostics, Switzerland). β-actin was used as the internal reference gene. Relative mRNA expression levels for each target gene were calculated using the 2^−ΔΔCt^ method. Primer sequences used in this study are listed in Supplementary Table S1.

### Western blot analysis

The tissues were minced and homogenized in RIPA lysis buffer to extract proteins. The lysates were centrifuged at 12,000 rpm for 20 min at 4 °C, and the supernatant containing soluble proteins was collected. Protein concentrations were quantified using a BCA protein assay kit. Equal amounts of protein lysates (approximately 30 μg per lane) were loaded and separated under reducing conditions on 15% (for hepcidin) or 10% (for other proteins) SDS–polyacrylamide gels. Following electrophoresis, proteins were transferred to PVDF membranes. The membranes were blocked with 10% bovine serum albumin (BSA) and then incubated overnight at 4 °C with the following primary antibodies: anti-TfR1 (1:1000), anti-DMT1 (1:1000), anti-Fpn1 (1:1000), anti-FTL (1:1000), anti-FTH (1:1000), anti-IRP1 (1:1000), anti-IRP2 (1:1000), anti-Nrf2 (1:1000), and anti-hepcidin (1:200), to enable specific binding to target proteins. Subsequently, the membranes were incubated with a goat anti-rabbit secondary antibody (1:5000) at 37 °C for 1 h. The intensities of the specific bands were detected and analyzed with the Odyssey infrared imaging system (Li-Cor, Lincoln, NE, USA).

### Statistical analysis

Statistical analyses were performed using GraphPad Prism 8.0. The results are presented as the mean ± standard error of the mean (S.E.M). The normality of the data distribution for inter-group comparisons was verified using the Shapiro–Wilk test. For data conforming to a normal distribution, an unpaired two-tailed t-test was employed, with a *P* value < 0.05 considered statistically significant. If the data do not conform to a normal distribution, nonparametric tests will be used.

## Results

### GPS ameliorates hepatic and splenic iron accumulation in ApoE-deficient mice

**Fig. 1:**
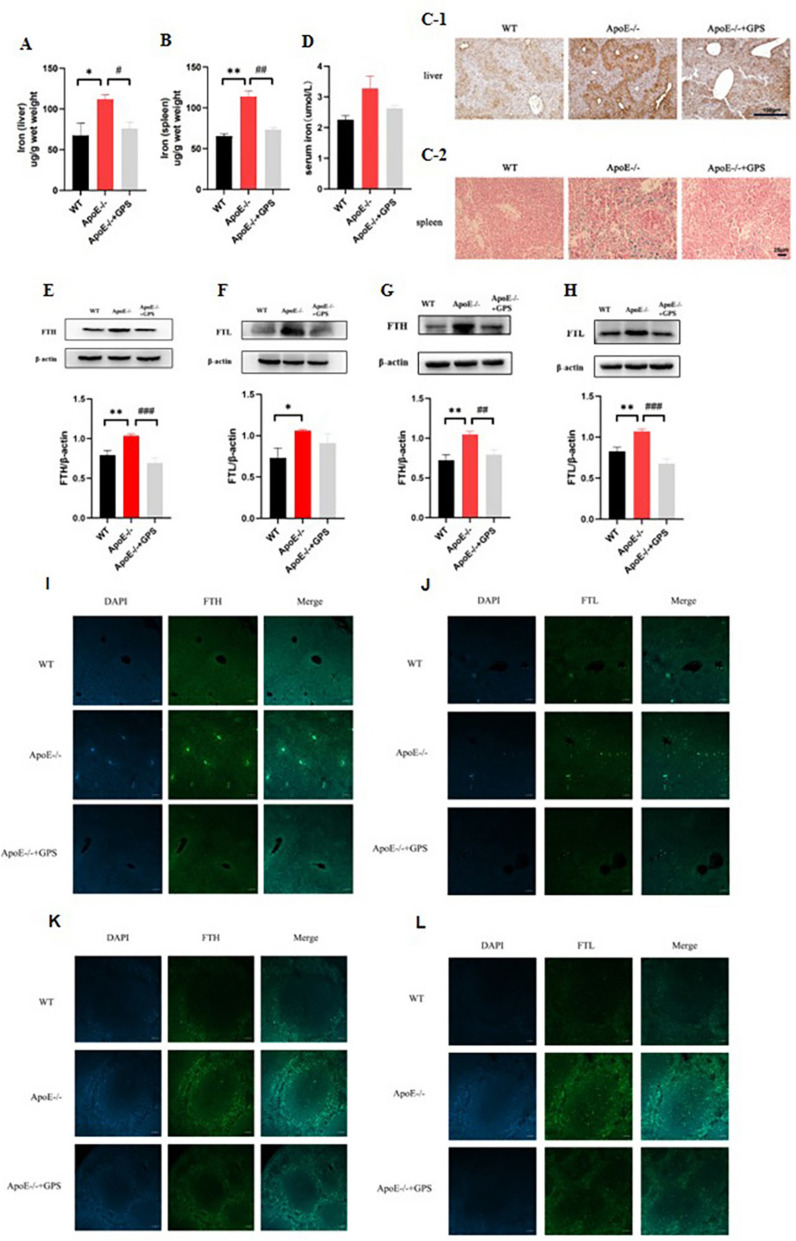
GPS Ameliorates Tissue Iron Accumulation in ApoE-Deficient Mice. Mice in the WT, *ApoE*^*−/−*^, and *ApoE*^*−/−*^ + GPS groups were handled as described in “Methods”. **A** and **B** Measurement of total iron contents in the liver (**A**) and spleen (**B**) of *ApoE*^*−/−*^ mice; **C**-1 DAB-Enhanced Perls Staining (Scale bar = 100 μm); **C**-2 Perl’s Iron Stain (Scale bar = 25 μm); **D** serum iron. **E** and **F** Expression of FTH protein(**E**) and FTL protein(**F**) in liver; **G** and **H** Expression of FTH protein(**G**) and FTL protein(**H**) in spleen; Immunofluorescence detection of FTH(**I**) and FTL(**J**) in the liver; Immunofluorescence detection of FTH(**K**) and FTL(**L**) in the spleen; Scale bar in panels (**I**–**L**) = 50 µm. All group *n* = 6; WB: *n* = 3. Data presented as the means ± S.E.M. *p < 0.05, **p < 0.01, ***p < 0.001vs. WT. ^#^*p* < 0.05, ^##^*p* < 0.01, ^###^*p* < 0.001 vs. *ApoE*^*−/−*^

To demonstrate the effect of *ApoE* on iron in mice, we first studied the tissue iron content of liver and spleen and serum iron (SI) in mice. Due to the difference in iron content between liver and spleen tissues, “DAB-Enhanced Perls Staining” and “Perl’s Iron Stain” were used with different scales for observation (the former is applied to visualize the overall iron distribution in the tissues, while the latter focuses on local iron deposits). The results confirmed that *ApoE* deficiency resulted in an increase in liver and spleen tissue iron, and a significant decrease in liver and spleen tissue iron content occurred in *ApoE*^*−/−*^ mice treated with GPS (Fig. 1A–C). However, there was no significant change in serum iron (Fig. 1D). In addition, Western blot analysis analysis and immunofluorescence detection of FTH and FTL in WT, *ApoE*^*−/−*^ and *ApoE*^*−/−*^ + GPS groups also confirmed that *ApoE* deficiency induced an increase in iron content in the liver (Fig. 1E–F, I–J) and spleen (Fig. 1G–H, K–L) of mice, and the iron content decreased significantly after GPS treatment. These results suggest that GPS may regulate iron metabolism in *ApoE*-deficient mice.

### GPS restores impaired iron transport in ApoE-deficient mice by improving TfR1 and Fpn1 expression

**Fig. 2:**
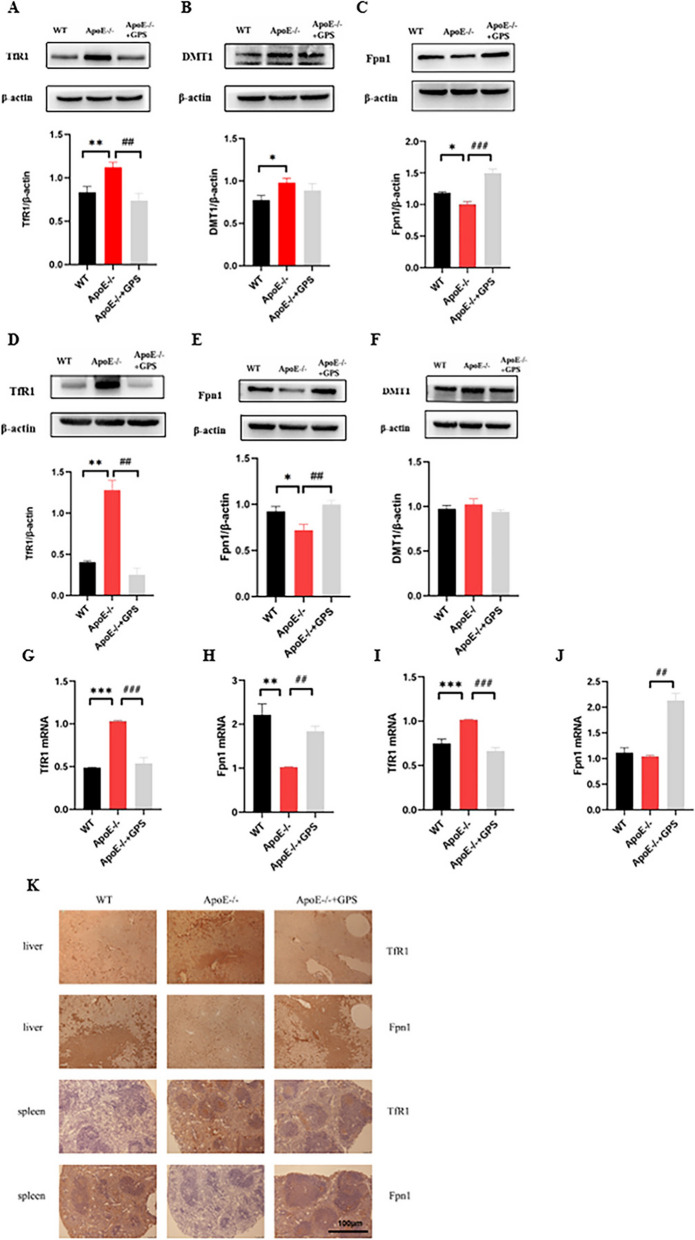
GPS ameliorates *ApoE* deficiency-induced dysregulation of TfR1 and Fpn1 expression in mice without affecting the upregulation of DMT1. Mice in the WT, *ApoE*^*−/−*^, and *ApoE*^*−/−*^ + GPS groups were handled as described in “Methods”. **A**–**C** Western blot analysis of TfR1, DMT1 and Fpn1 expression in the liver; **D**–**F** Western blot analysis of TfR1, Fpn1 and DMT1 expression in the spleen; **G**–**H** RT-PCR analysis of TfR1 mRNA and Fpn1 mRNA in the liver; **I**–**J** RT-PCR analysis of TfR1 mRNA and Fpn1 mRNA in the spleen; **K** Immunohistochemical examination of TfR1 and Fpn1 protein expression in the liver and spleen (Scale bar = 100 μm). All group *n* = 6; WB: *n* = 3. Data presented as the means ± S.E.M. *p < 0.05, **p < 0.01, ***p < 0.001vs. WT. ^#^*p* < 0.05, ^##^*p* < 0.01, ^###^*p* < 0.001 vs. *ApoE*^*−/−*^

To investigate the mechanism by which GPS affects tissue iron contents, we investigated the effect of *ApoE* deficiency on TfR1, DMT1 and Fpn 1 protein expression. Because TfR1, DMT1 and Fpn1 are key proteins that regulate cellular or tissue iron levels [27, 28]. Western blot analysis showed that in the liver, The expression of TfR1 (Fig. 2A) and DMT1 (Fig. 2B) are significantly increased and Fpn1 (Fig. 2C) expression was significantly decreased in *ApoE*^*−/−*^ mice compared with WT mice, while the protein expression of TfR1 (Fig. 2A) and Fpn1 (Fig. 2C) was reversed in *ApoE*^*−/−*^ mice after GPS treatment (The expression of TfR1 protein was decreased, and the expression of Fpn1 protein was significantly increased), but no significant change in DMT1 protein expression (Fig. 2B). In the spleen, ApoE deficient mice also had a significant increase in TfR1 protein expression (Fig. 2D) and a significant decrease in Fpn1 protein expression (Fig. 2E) compared with WT mice. Moreover, the expression of TfR1 and Fpn1 was reversed in *ApoE*^*−/−*^ mice after GPS treatment. And there was no significant change in DMT1 expression (Fig. 2F). In RT-PCR analysis, TfR1 mRNA expression was significantly increased and Fpn1 mRNA expression was decreased in liver (Fig. 2G–H) and spleen (Fig. 2I–J), and their expression was reversed after GPS treatment. Furthermore, immunohistochemical examination of TfR1 and Fpn1 protein expression in the liver and spleen (Fig. 2K) yielded consistent results.

### GPS modulates systemic iron regulation by tissue-specific effects on hepcidin and IRPs

**Fig. 3:**
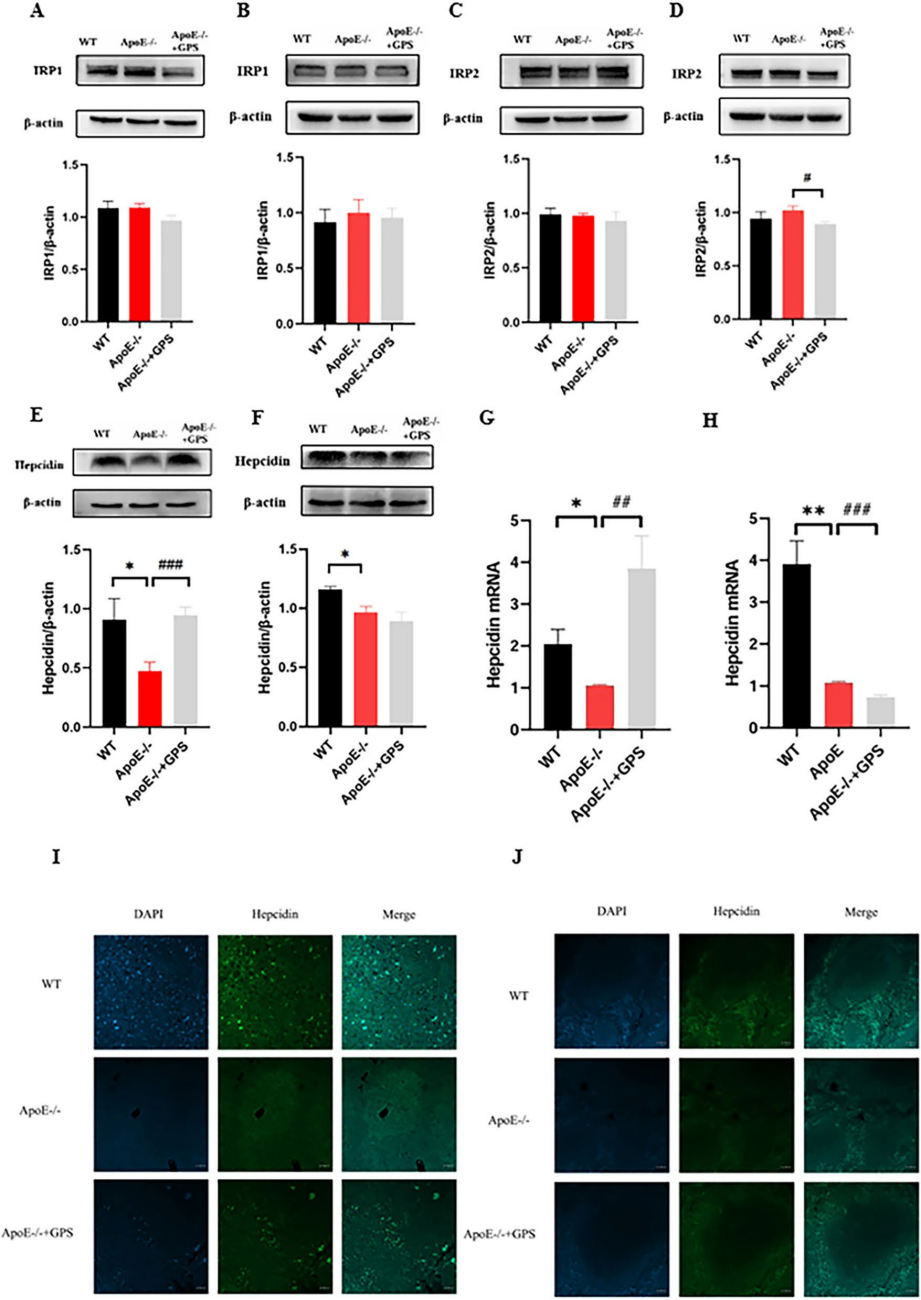
GPS Exhibits Tissue-Specific Regulation of Systemic Iron Homeostasis in ApoE-Deficient Mice. Mice in the WT, *ApoE*^*−/−*^, and *ApoE*^*−/−*^ + GPS groups were handled as described in “Methods”. **A**–**F** Western blot analysis of IRPs and hepcidin in the liver (**A**, **C** and **E**) and spleen (**B**, **D** and **F**). **G** and **H** RT-PCR analysis of hepcidin mRNA in the liver (**G**) and spleen (**H**); **I** and **J** Immunofluorescence examination of hepcidin protein in liver (**I**) and spleen (**J**) (Scale bar = 50 μm). All group *n* = 6; WB: *n* = 3. Data presented as the means ± S.E.M. *p < 0.05, **p < 0.01, ***p < 0.001vs. WT. ^#^*p* < 0.05, ^##^*p* < 0.01, ^###^*p* < 0.001 vs. *ApoE*^*−/−*^

Cellular iron homeostasis is coordinated by the Iron Regulatory Elements (IREs) and iron-responsive protein (IRP) system, which modulates TfR1, DMT1, and the expression of both chains of ferritin and the iron exporter Fpn1. Additionally, the hepcidin-ferroportin regulatory system is involved in regulating iron release from macrophages, enterocytes, and a multitude of other cells, thus influencing systemic iron homeostasis [29, 30]. To ascertain the impact of *ApoE* deficiency on TfR1 and Fpn1 expression, we proceeded to examine the influence of *ApoE* deficiency on IRP and hepcidin expression in mice. Western blot analysis demonstrated that there was no significant difference in the expression of IRPs (IRP1 (Fig. 3A–B) and IRP2 (Fig. 3C–D)) between *ApoE*^*−/−*^ mice and wild type mice, indicating that the changes of TfR1 and Fpn1 expression induced by *ApoE*^*−/−*^ mice may not be related to IRP. This conclusion was also confirmed by immunofluorescence examination. We also demonstrated that hepcidin expression in the liver (Fig. 3E, G, I) and spleen (Fig. 3F, H, J) of *ApoE*-deficient mice was significantly lower than that in WT mice as determined by WB, RT-PCR and immunofluorescence. The results for Fpn1 suggested that *ApoE*^*−/−*^ mice would induce a decrease in hepcidin expression, while theoretically, a decrease in Fpn1 expression should be accompanied by an increase in hepcidin expression [31, 32]; however, the experimental results showed a decrease in hepcidin expression in both liver and spleen of *ApoE*^*−/−*^ mice, rather than a theoretical increase. This might imply that hepcidin does not play a predominant role in the control of Fpn1 expression in *ApoE*^*−/−*^ mice. In addition, we found that the tissue iron content of GPS treated *ApoE*^*−/−*^ mice was significantly decreased. To further find out the reason, we also detected the protein expression of IRPs and hepcidin. The results showed that there were differences in the expression of IRPs and hepcidin proteins in the liver and spleen of *ApoE*^*−/−*^ mice treated with GPS. In liver (Fig. 3A, C, E, G, I), hepcidin protein expression was significantly increased, and IRPs had no significant change. However, the expression of hepcidin and IRP2 in spleen (Fig. 3B, D, F, H, J) was significantly decreased, and the RT-PCR and immunofluorescence follow the same trend. This may indicate that GPS does not reduce liver and spleen iron content by exactly the same mechanism in *ApoE*-deficient mice, and the results may suggest that down-regulated expression of IRP2 and hepcidin may play an important role in GPS-induced decrease in spleen TfR1 expression and increase in spleen Fpn1 expression in *ApoE*^*−/−*^, whereas it does not play a major role for the liver.

### GPS activates the Nrf2 pathway in a spleen-selective manner to mitigate iron dysregulation in ApoE-deficient mice

**Fig. 4:**
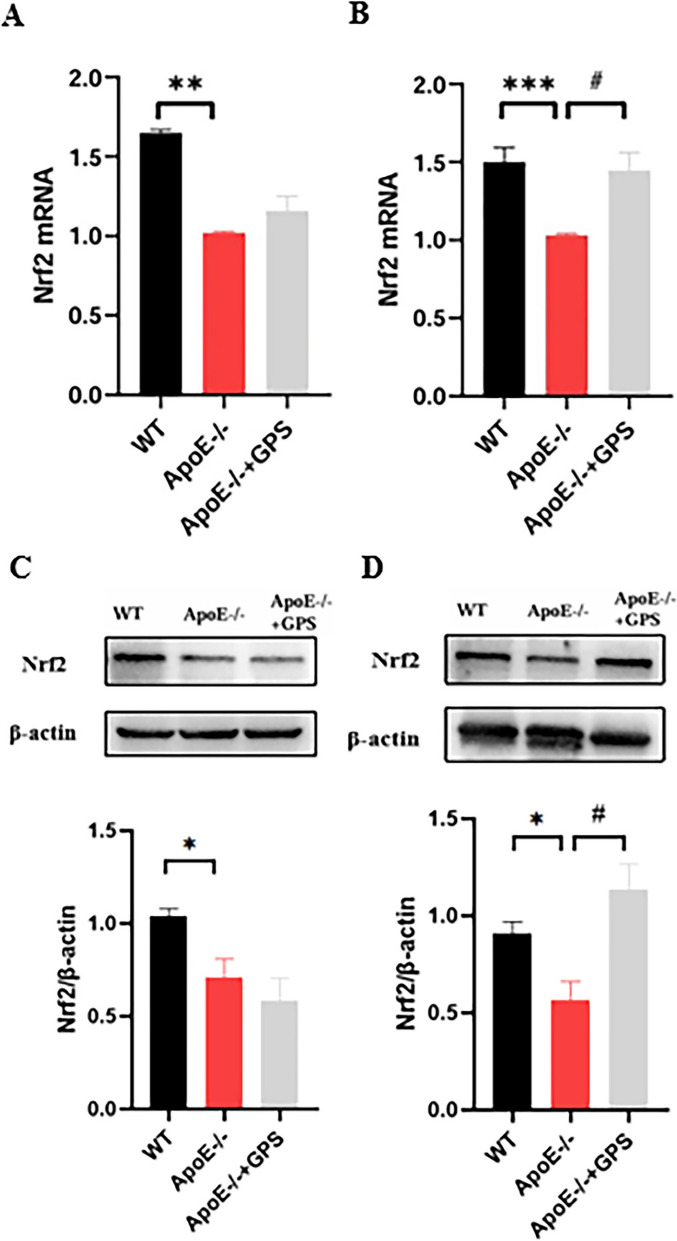
GPS Activates the Nrf2 Pathway in a Spleen-Selective Manner in ApoE-Deficient Mice. Mice in the WT, *ApoE*^*−/−*^, and *ApoE*^*−/−*^ + GPS groups were handled as described in “Methods”. **A**–**B** RT-PCR analysis of Nrf2 mRNA in the liver (**A**) and spleen (**B**); Western blot analysis of Nrf2 in the liver (**C**) and spleen (**D**). All group *n* = 6; WB: *n* = 3. Data presented as the means ± S.E.M. *p < 0.05, **p < 0.01, ***p < 0.001vs. WT. ^#^*p* < 0.05, ^##^*p* < 0.01, ^###^*p* < 0.001 vs. *ApoE*^*−/−*^

To further explore the mechanism by which Fpn1 expression is down-regulated by *ApoE* deficiency, we investigated the mRNA and protein expression of Nrf2 in liver and spleen, as NRF2 has been reported to be an iron signal transduction regulator, which regulates Fpn1 expression to maintain iron homeostasis [33, 34]. The results of the study showed that the Nrf2 mRNA expression was significantly lower in the liver (Fig. 4A) and spleen (Fig. 4B) of *ApoE*^*−/−*^ mice than in WT mice. Protein expression of Nrf2 was also significantly reduced in the liver (Fig. 4C) and spleen (Fig. 4D) of *ApoE*^*−/−*^ mice compared with WT mice. On the other hand, Nrf2 mRNA and protein expression were significantly increased in the spleen of GPS-treated *ApoE* KO mice, while no significant changes were observed in the liver. This imply that Nrf2-mediated down-regulation of Fpn1 is the main reason for the increase of iron in the liver and spleen of *ApoE* KO mice, and GPS can reduce iron accumulation in the spleen of *ApoE* KO mice by up-regulating the expression of Nrf2/Fpn1, but this pathway has no significant effect in the liver.

## Discussion

In this paper, we first demonstrated that *ApoE* deficiency led to increased iron content and elevated expression of FTH and FTL proteins in the liver and spleen tissues of mice. Notably, GPS treatment effectively reduced iron accumulation and ferritin expression in these tissues, indicating that *ApoE* is essential for maintaining systemic iron homeostasis and that GPS mitigates iron overload in *ApoE*-deficient mice. To elucidate the mechanisms underlying iron accumulation in *ApoE*^*−/−*^ mice, we investigated the expression of TfR1 and Fpn1, as iron balance in most cell types depends on the normal expression of these iron influx and efflux transporters [32, 35, 36]. Compared to WT mice, *ApoE*^*−/−*^ mice exhibited upregulated TfR1 mRNA and protein expression alongside downregulated Fpn1 expression in both liver and spleen, suggesting that *ApoE* deficiency enhances cellular iron uptake (via TfR1 upregulation) while suppressing iron export (via Fpn1 downregulation). Thus, dysregulation of TfR1 and Fpn1 represents a critical mechanism driving iron accumulation in *ApoE*^*−/−*^ mice. Intriguingly, GPS treatment reversed these alterations, highlighting its potential to restore iron homeostasis by modulating TfR1 and Fpn1 expression. A key question arising from these findings is how *ApoE* deficiency disrupts TfR1 and Fpn1 expression and how GPS counteracts these effects. Previous studies have established that TfR1 and Fpn1 expression is primarily regulated by IRPs, hepcidin, and Nrf2 [37–39]. We therefore analyzed these regulatory pathways in detail.

i. Hepcidin/Fpn1 Axis. Systemic iron homeostasis is regulated at two levels: systemic (via the hepcidin/Fpn1 axis) and cellular (via IRP/IRE signaling) [40]. At the systemic level, iron homeostasis is regulated through the hepcidin/FPN axis [41]. Hepcidin directly binds to Fpn1, reducing the amount of Fpn1 on the cell membrane. Subsequently, the hepcidin/Fpn1 complex is internalized and degraded [42–45]. An increase in hepcidin levels may lead to a decrease in Fpn1, and it has been demonstrated that hepcidin treatment can reverse iron overload in the brains of iron-overloaded rats by inhibiting the expression of ferroportin [46, 47]. In this study, when comparing *ApoE*^*−/−*^ mice with WT mice, we found that *ApoE* deficiency led to a significant decrease in the expression of hepcidin in the liver and spleen of mice. However, our experimental result shows a downregulation of hepcidin expression, which is contrary to our theoretical understanding, that is, the downregulation of Fpn1 can negatively feedback to increase hepcidin. The reason for this contradiction is considered to be that the decrease in hepcidin expression may be due to the increased iron content in the organs induced by *ApoE*^*−/−*^. Because hepcidin has been shown to have an inhibitory effect on the expression of TfR1 in different types of cells [31, 48, 49]. Therefore, it is inferred that the overexpression of TfR1 caused by *ApoE* deficiency may be at least partially attributed to the downregulation of hepcidin. These unexpected findings suggest that hepcidin may not play a dominant role in the *ApoE* KO-induced alterations of TfR1 and Fpn1 expression under our experimental conditions. Furthermore, GPS treatment downregulated hepcidin expression in the spleen of *ApoE*^*−/−*^ mice. This implies that GPS may reduce iron accumulation by enhancing iron efflux via hepcidin-dependent mechanisms. Hepcidin is not only regulated by the systemic iron level but also affected by inflammation. For instance, inflammation induces the expression of IL-6 and activin B in the liver, which activates the transcription of hepcidin via the STAT3/JAK2 pathway and the BMP signaling pathway, respectively [50]. While *ApoE* knockout induces systemic iron overload—a condition known to provoke inflammatory responses—there is insufficient evidence to determine whether *ApoE* deficiency-driven iron accumulation reaches a threshold sufficient to trigger inflammatory feedback mechanisms. This constitutes a key focus of our ongoing research.
ii. Nrf2/Fpn1 Axis. Nrf2, a key regulator of iron signaling, modulates Fpn1 expression to maintain iron homeostasis. Our data indicate that *ApoE* deficiency significantly reduces the mRNA and protein levels of Nrf2 in the liver and spleen of mice. Moreover, we have discovered an interesting phenomenon: after GPS treatment, the expression of Nrf2 in the spleen of *ApoE-/-* mice increases significantly, while no obvious change is observed in the liver. This may indicate that, in the Nrf2, GPS upregulates the expression of Nrf2 and Fpn1 in the spleen, and it may reduce iron accumulation through this pathway, but further verification is still required via Nrf2 inhibitor or gene intervention experiments. The reason for this organ-specific difference may be the specificity of iron metabolism regulation among different organs. There is evidence that splenic macrophages recycle approximately 20 mg of iron per day, accounting for 90% of the systemic circulating iron [51], and the regulatory mechanisms of iron metabolism in the two organs are different. The liver regulates the systemic iron distribution through hepcidin, while iron recycling in the spleen mainly depends on the expression of Fpn1 in macrophages [52]. The direct association between Nrf2 and Fpn1 in macrophages has been clearly established [53]. In addition, GPS has been confirmed by various experiments to have the effects of activating antioxidant enzymes, inhibiting inflammatory pathways, and protecting cells. Some studies have proven that GPS can significantly improve the oxidative stress status of the liver and spleen by activating the Nrf2 pathway, inhibiting NF-κB inflammatory response, and directly scavenging free radicals [54–56]. Iron accumulation can induce oxidative stress and stimulate the release of inflammatory factors, which have adverse effects on the body. Currently, GPS has been confirmed to have the pharmacological efficacy of anti-oxidative stress and reducing the release of inflammatory factors (such as IL-6 and TNF-α) [1, 3]. Based on this background, it can also explain why GPS can reverse the expressions of TfR1 and Fpn1 in the tissues of *ApoE*^*−/−*^ mice.
iii. IRP/IRE Axis. Cellular iron homeostasis is tightly regulated by IRPs (IRP1 and IRP2), which post-transcriptionally control the expression of iron-related proteins (e.g., TfR1, DMT1, Fpn1, ferritin) via IRE binding [57–59]. We have demonstrated that *ApoE* knockout had no effect on the expressions of IRP1 and IRP2 in the liver and spleen of the mice in our study. This indicates that *ApoE* knockout does not affect the regulation of iron by IRP. However, interestingly, after treatment with GPS, we found that the protein expression of IRP2 in the spleen of *ApoE*^*−/−*^mice was significantly reduced, while there was no obvious change in the liver. For this phenomenon, currently, we can only speculate that the reduction of iron in the spleen by GPS may be related to the regulation of IRP2. But why there is no such manifestation in the liver, the relevant mechanism is still unclear, and there is no relevant literature report, which still requires further investigation.

In addition, it was detected that in the liver and spleen of *ApoE*^*−/−*^ mice, although TfR1 and Fpn1 showed significant changes, the protein expression of DMT1, which is also an iron transporter, did not exhibit any alteration. We speculate that this is due to tissue specificity. Evidence indicates [60] that the expression of DMT1 is the highest in the duodenum of iron-deficient animals, and most types of cells use the Tf-TfR1-mediated pathway to uptake iron into the cytosol. As the major iron importer, DMT1 is mainly regulated at the translational level through IRE-IRP binding. In this study, no significant changes in IRPs were detected in the liver and spleen of *ApoE*^*−/−*^ mice, which is presumably one of the reasons why no obvious change in DMT1 expression was found. Additionally, studies have confirmed [61] that the expression of DMT1 varies in different tissues, and in some tissues (such as the liver and kidney), DMT1 can only be expressed after translational or post-translational up-regulation.

Although this study provides the first evidence for GPS-mediated regulation of iron metabolism, several limitations must be acknowledged. First, our experimental model was confined to mice. Whether the observed iron-modulatory effects of GPS in *ApoE*^*−*^*/*^*−*^ mice translate to humans—particularly given human *ApoE* allelic heterogeneity and inflammation-driven iron dysregulation—requires validation in human-derived models or clinical studies. Furthermore, given that there is currently no published evidence regarding GPS-mediated iron regulation, the dosage and intervention duration of GPS in this study can only be referenced from its pharmacological effects in lipid metabolism. Therefore, this is a preliminary proof-of-concept study. Consequently, a limited cohort size was employed at this initial stage. Future research will expand sample sizes and examine additional iron-regulatory pathways based on these foundational findings. Another limitation is the lack of functional manipulations to establish causal relationships (e.g., Nrf2 inhibitors, recombinant hepcidin) and supporting cellular experiment evidence (e.g., construction of HepG2 and RAW264.7 cell models), and clarification of precise subcellular localization. Although our data indicate that key iron regulatory factors are altered following GPS treatment, mechanism validation warrants future interventional studies.

## Conclusions

In conclusion, our study provides the first evidence that GPS reduces iron accumulation in the liver and spleen of *ApoE*^*−/−*^ mice by modulating the expression of cellular iron transporters TfR1 and Fpn1, albeit through organ-specific mechanisms. In the spleen, iron reduction likely involves Nrf2-dependent upregulation of Fpn1 and IRP2-mediated downregulation of TfR1. In contrast, hepatic iron reduction appears independent of Nrf2 and IRPs based on current findings, suggesting distinct regulatory pathways in the liver. The paradoxical relationship between hepcidin and Fpn1 may stem from *ApoE* deficiency-induced alterations in tissue iron content, with TfR1 upregulation partially contributing to this phenomenon. However, the precise mechanisms governing hepatic iron metabolism in this context remain incompletely elucidated and are the focus of ongoing investigations in our laboratory. Iron overload-related pathologies are increasingly prevalent, and our findings highlight GPS as a promising therapeutic candidate for iron metabolism disorders. Further exploration of the molecular underpinnings of iron regulation, particularly the organ-specific networks identified here, will deepen mechanistic understanding and inform targeted therapeutic strategies.

## Supplementary Information

Supplementary Material 1. Targeted disruption of the mouse ApoE.

Supplementary Material 2. The specific pairs of primers.

## Data Availability

All data are contained within the manuscript.

## Abbreviations

ApoE: Apolipoprotein E
ApoE^−^/^−^: Apolipoprotein E knock-out
ADH: Autosomal dominant hypercholesterolemia
DMT1: Divalent metal transporter 1
Fpn1: Ferroportin 1
FTH: Ferritin heavy chain
FTL: Ferritin light chain
GFAAS: Graphite furnace atomic absorption spectrophotometer
GP: *Gynostemma pentaphyllum*
GPS: Gypenosides
GPP: Polysaccharides
IRPs: Iron regulatory proteins
SI: Serum iron
TfR1: Transferrin receptor 1
WT: Wild-type
VLDL: Very-low-density lipoprotein
