# Supplementary material for: Effect of saponins from gynostemma pentaphyllum on iron metabolism in apolipoprotein E deficient mice
Source: Eur J Med Res. 2026 Jan 12;31:260. doi: 10.1186/s40001-026-03871-6 (PMC12888491; doi:10.1186/s40001-026-03871-6)
Supplement: Supplementary file 1 — Supplementary Material 1. Targeted disruption of the mouse ApoE. [file 40001_2026_3871_MOESM1_ESM.docx]

**Fig. S1** **Targeted disruption of the mouse ApoE**

PCR-genotyping of ApoE knockout mice was performed using a two-primer assay in onereaction.

Primer primer

ApoE F TGCCTAGTCTCGGCTCTGAACTAC

ApoE R CAACCTGGGCTACACACTAATTGAG

ApoE^-/-^ mice product size is 346bp, ApoE^+/+^ mice produce size is 428bp.


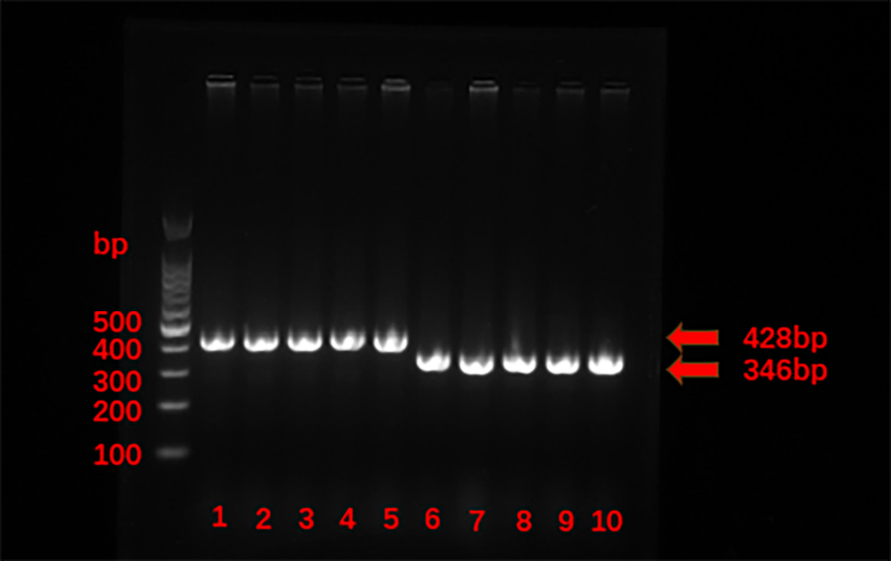


The ApoE KO mice we used were all offspring of ApoE-/- bred to ApoE-/-, and random sampling was verified by PCR. The results showed that the number of 1-5 were wide type, while 6-10 were ApoE KO.
