# Supplementary material for: Effect of saponins from gynostemma pentaphyllum on iron metabolism in apolipoprotein E deficient mice
Source: Eur J Med Res. 2026 Jan 12;31:260. doi: 10.1186/s40001-026-03871-6 (PMC12888491; doi:10.1186/s40001-026-03871-6)
Supplement: Supplementary file 2 — Supplementary Material 2. The specific pairs of primers. [file 40001_2026_3871_MOESM2_ESM.docx]

**Table. S1 The specific pairs of primers**

| Primers | Primer sequence |
| --- | --- |
| β-actin forward | 5’-AAATCGTGCGTGACATCAAAGA-3’ |
| β-actin reverse | 5’-GGCATCTCCTGCTCGAAGTC-3’ |
| m-Hepcidin forward | 5’-GGCAGACATTGCGATACCAA-3’ |
| m-Hepcidin reverse | 5’-GAAGATGCAGATGGGGAAGT-3’ |
| m-TfR1 forward | 5’-TGGGTCTAAGTCTACAGTGGC-3’ |
| m-TfR1 reverse | 5’-AGATACATAGGGCGACAGGAA-3’ |
| m-Fpn1 forward | 5’-TCACCTGGCTACGTCGAAAAT-3’ |
| m-Fpn1 reverse | 5’-GCTGGGCTAGTCCTGAGAATAGAC-3’ |
| m-Nrf2 forward | 5’-CAGTGCTCCTATGCGTGAA-3’ |
| m-Nrf2 reverse | 5’-GCGGCTTGAATGTTTGTC-3’ |
